# Validating reference genes using minimally transformed qpcr data: findings in human cortex and outcomes in schizophrenia

**DOI:** 10.1186/s12888-016-0855-0

**Published:** 2016-05-20

**Authors:** Brian Dean, Madhara Udawela, Elizabeth Scarr

**Affiliations:** The Florey Institute for Neuroscience and Mental Health, Parkville, VIC Australia; The Department of Psychiatry, the University of Melbourne, Victoria, Australia; The Division of Biological Psychiatry and Mental Health and the Molecular Psychiatry Laboratory, The Florey Institute for Neuroscience and Mental Health, 30 Royal Parade, Parkville, VIC 3052 Australia

**Keywords:** Schizophrenia, Cortex, Post-mortem CNS, qPCR, Reference genes, mRNA

## Abstract

**Background:**

It is common practice, when using quantitative real time polymerase chain reaction (qPCR), to normalise levels of mRNA to reference gene mRNA which, by definition, should not vary between tissue, with any disease aetiology or after drug treatments. The complexity of human CNS means it unlikely that any gene could fulfil these criteria.

**Methods:**

To address this issue we measured levels of mRNA for six potential reference genes (*GAPDH*, *PPIA, SNCA, NOL9, TFB1M and SKP1*) in three cortical regions (Brodmann’s areas (BA) 8, 9 and 44) from 30 subjects with schizophrenia and 30 age and sex matched controls. We used a structured statistical approach to examine the characteristics of these data to determine their suitability as reference genes. We also analysed our data using reference genes selected by rank as defined using the average of the standard deviation of pair-gene ΔCt and the BestKeeper, NormFinder and geNorm algorithms to determine if they suggested the same reference genes.

**Results:**

Our minimally derived data showed that levels of mRNA for all of the six genes varied between cortical regions and therefore no gene fulfilled the absolute requirements for use as reference genes. As levels of some mRNA for some genes did not vary with diagnoses within a cortical region from subjects with schizophrenia compared to controls, we normalised levels of mRNA for all the other genes to mRNA for one, two or three reference genes in each cortical region. This showed that using the geometric mean of at least two reference genes gave more reproducible results. Finally, using the reference gene ranking protocols the average of the standard deviation of pair-gene ΔCt, BestKeeper, NormFinder and geNorm we showed that these approaches ranked potential reference genes differently. We then showed that outcomes of comparing data from subjects with schizophrenia and controls varied depending on the reference genes chosen.

**Conclusions:**

Our data shows that the selection of reference genes is a significant component of qPCR study design and therefore the process by which reference genes are selected must be clearly listed as a potential confound in studying gene expression in human CNS. This should include showing that, using minimally derived qPCR data, levels of mRNA for proposed reference genes does not vary with variables such as diagnoses and CNS region.

**Electronic supplementary material:**

The online version of this article (doi:10.1186/s12888-016-0855-0) contains supplementary material, which is available to authorized users.

## Background

Quantitative real time polymerase chain reaction (qPCR) is widely used to measure levels of mRNA in biological samples. As qPCR aims to quantify mRNA in tissue, variables such as the efficiencies of extracting RNA and differences in the synthesis of cDNA from RNA in different samples could be significant experimental confounds. It is now a wide spread practice to normalise data to levels of mRNA from a gene designated as a reference gene [1]; a reference, or housekeeping, gene being a gene that gives levels of mRNA that do not vary between different biological samples, are not affected by any experimental procedure or disease aetiologies [2]. Such stable expression properties should mean that normalising levels of less stable genes to the reference gene controls for confounds such as tissue mRNA extraction and variability in cDNA synthesis across different samples. However, it has long been recognised that finding genes that meet the precepts of a reference gene is a significant challenge [3].

The complexity of the central nervous system (CNS) means it is a particularly difficult organ in which to find genes with expression characteristics consistent with that of a reference gene. Indeed, current data suggests that finding genes with expression levels that do not vary between CNS region and disease state may not be feasible [4–6]. Similarly, there is a question as to whether there are genes that can be designated reference genes in studies comparing gene expression in CNS and peripheral tissue [7]. To tackle these problems attempts have been made to develop algorithms that can rank genes in order of suitability for use as a reference gene using data from qPCR [5]. Unfortunately, given the same data sets, different algorithms will suggest different reference genes and thus study outcomes can vary depending on the algorithms used to select reference genes [1].

It is now widely accepted that psychiatric disorders such as schizophrenia occur in individuals with a genetic predisposition after they have encountered environmental triggers that lead to the frank onset of illness [8]. The impact of the interaction between genes and environment occurs through epigenetic mechanisms that bring about changes in CNS gene expression [9] and therefore measuring gene expression in the CNS of subjects with psychiatric disorders has been used to gain insight into their aetiologies [10]. However, it is now increasingly recognised that the study of gene expression in CNS from subjects with psychiatric disorders such as schizophrenia is hampered by problems in identifying suitable reference genes for data normalisation [11, 12]. It has been suggested that problems with variability in reference genes in post-mortem CNS from subjects with psychiatric disorders can be overcome by using the geometric mean of levels of three reference genes [13]. However, this solution is still dependent on being able to identify reference genes with appropriate expression characteristics.

We have addressed the issue of identifying reference genes by examining levels of mRNA for three commonly used reference genes (glyceraldehyde-3-phosphate dehydrogenase (*GAPDH*), peptidylprolyl isomerase A (cyclophilin A) (*PPIA*), synuclein, alpha (non A4 component of amyloid precursor) (*SNCA*) in three human cortical regions. We also measured mRNA levels of three genes, nucleolar protein 9 (*NOL9*), transcription factor B1, mitochondrial (*TFB1M*) and S-phase kinase-associated protein 1 (*SKP1*)), that we had identified as potential reference genes from publically available microarray data from human CNS using the Genevestigator platform [14].

Whilst practical issues support expressing data from qPCR as a ratio of one or more reference genes, the transformation of data into ratios introduces inherent problems in subsequent statistical analyses [15–17]. This is because i) there may not be a linear relationship between the numerator and denominator, ii) if there is not a zero intercept between the numerator and denominator this will make the measure of the denominator a confounding effect, iii) minor departures from a zero intercept can have major consequences on the ratio’s ability to control for the denominator, iv) the use of ratios cannot easily take nonlinear effects between the numerator and denominator into account, v) the use of ratios can introduce spurious correlations among the ratios and other variables, vi) the use of ratios can create interpretive difficulties and importantly vi) the use of ratios affects the error distribution of the data which may also violate the assumptions of subsequent parametric statistical analyses. Hence, to attempt to avoid analysing data in the form of ratio to identify potential reference genes we used a relatively underived data to identify characteristics in gene expression that best fit with a gene suitable for use as a reference gene. We then compared genes we identified as potential reference genes to outcomes suggested by other approaches using our data from subjects with schizophrenia with respect to controls. The other approaches to identifying reference genes included using the average of the standard deviation of pair-gene ΔCt [18] and three published algorithms (BestKeeper [19], NormFinder [20] and geNorm [21]) that have been developed to rank genes in order of their potential for use a reference genes.

## Methods

### Human CNS collection

Approval to collect tissue used in these studies was obtained from the Ethics Committee of the Victorian Institute of Forensic Medicine and the Mental Health Research Institute. Tissue was collected following consent been gained from the next-of-kin and supplied by the Victorian Brain Bank Network.

For these studies, tissue was collected from Brodmann’s area (BA) 8 (defined as being primarily in the superior frontal gyrus and extending from the cingulate sulcus on the medial surface to the middle frontal gyrus), 9 (the lateral surface of the frontal lobe including the middle frontal gyrus superior to the inferior frontal sulcus) and 44 (the region occupying the opercular region of the inferior frontal gyrus, bounded rostrally by the ascending limb of the lateral sulcus and caudally by the inferior precentral sulcus) from the left hemisphere from 30 subjects who had suffered from schizophrenia and 30 age and sex matched subjects with no history of psychiatric illness (controls). A case history review was completed using the Diagnostic Instrument for Brain Studies [22, 23]. During the case history review suicide completion was recorded when listed in the Coroner’s report. Duration of illness (DI) was calculated as time from first clinical presentation to a psychiatric service to death. The final recorded dose of antipsychotic drug (FRADD) was recorded and converted to chlorpromazine equivalents using algorithms as proposed in the literature [24], as was total lifetime exposure to such drugs (LEAP). On completion of the case history review diagnoses were made according to DSM-IV criteria by consensus between a psychologist and a senior psychiatrist.

All cadavers were refrigerated within 5 h of being found. When death was witnessed, post-mortem interval (PMI) was calculated as the time from death to autopsy. Where death was not witnessed, tissue was only collected from subjects who had been seen alive up to 5 h prior to being found dead; here the PMI was taken as the midpoint between the person being found and being last seen alive. As well as maintaining cases at low temperatures for most of their PMI, tissue was rapidly processed and frozen to -70 °C within 30 min of autopsy [25]; processing tissue in this way significantly slows autolytic changes [26]. CNS pH was measured as described previously [27] as this provides a good measure of overall tissue quality [28].

### RNA purification and first-strand cDNA synthesis

Total RNA was isolated from 100 mg frozen tissue using 1 mL TRIzol ® reagent (ThermoFisher Scientific, Waltham, MA USA) according to the manufacturer’s instructions. The RNA was treated with DNase I (ThermoFisher Scientific) at 37 ° C for 25 – 30 min and then purified by phenol/chloroform extraction and stored at -80 ° C. RNA quantity and quality were determined by spectrophotometer readings (NanoDrop, ThermoFisher Scientific) and RNA integrity numbers (RINs) obtained from an Agilent 2100 bioanalyser [29]. Genomic DNA elimination was confirmed by PCR using primers specific for genomic DNA. First strand cDNA was synthesized from 2 μg RNA using 100 units M-MLV-RT (ThermoFisher Scientific) with 2.5 μM random decamers and 2.5 μM oligo dT primers (ThermoFisher Scientific), 0.5 mM of each dNTP and 20 units RNase inhibitor in RT Buffer (50 mM Tris–HCl, pH 8.3, 75 mM KCl, 3 mM MgCl 2 and 5 mM DTT) in a final volume of 20 μL. The reaction was incubated at 44 ° C for 1 h then inactivated at 92 ° C and the product aliquoted and stored at -20 ° C.

### Real-time PCR assay

We chose 6 potential reference genes using 2 criteria. Levels of *GAPDH*, *PPIA* and *SNCA* were measured because they have been used widely as reference genes in the studies using human CNS [21, 30]; levels of *NOL9*, *TFB1M* and *SKP1* were measured because we had used these genes as reference genes in a previous study [31] having first selected them as potential reference genes from microarray data [32].

cDNA was used as a template for real-time PCR. Reactions were performed, in triplicate, in a Bio-Rad iQ5 Real-Time PCR Detection System with Bio-Rad iQ5 optical system 2.0 software (Bio-Rad, Hercules, CA) in 50 μL volume containing cDNA diluted 1:125, 0.4 nM primers and 1 x IQ SYBR green supermix (Bio-Rad), with cycling conditions of 95 ° C for 3 min, 40 cycles of 30 s each at 95 ° C, 57 ° C and 72 ° C, followed by a melt curve. Relative quantity was determined corrected for reaction efficiencies using the Pfaffl method [33]. Primers (Additional file 1: Table S1) were deemed acceptable if they gave reaction efficiencies, calculated from standard curves run on each plate constructed from a 10-fold dilution series of the human CNS cDNA, of between 90 and 110 % and were shown to amplify the expected gene according to the nucleotide sequence of their amplicon. The standard curves run for every gene on every plate were made from the same batch of cDNA and any minor plate to plate variation was corrected to a calibrator sample, prepared from a subject that was not part of the cohort, which was included on every plate. Analyses of melt curves showed there was no non-specific amplification or primer dimerization (Additional file 2: Figure S1).

### Statistics

Cohort age, PMI and CNS pH were compared between diagnoses using the Student’s *t*-test. Gender frequency was compared using a *χ*^2^ test. Relationships between age, PMI, DI, CNS pH, RIN, FRADD and LEAP and experimental data were determined using linear regression and the resulting coefficient of determination. The small sample sizes in this study meant that only strong relationships, r^2^ ≥ 0.49 [34], warranted further consideration as potential confound. Where there were strong correlations non-experimental data were included as covariates when analysing experimental data.

For “in house” analyses using raw and derived data we made minimal assumptions about the nature of the data. However, the qPCR data sets showed a complex mixture of parametric and non-parametric distribution and hence we used non-parametric analyses (Mann–Whitney *U* test or Kruskal-Wallis test) as these types of analyses best identify differences between such mixed data [35].

Another approach to identifying potential reference genes was to calculate the ΔCTs for each combination of potential reference gene pair in a single tissue sample, and compare the standard deviations of ΔCT for each gene pair across the sample set [18]. To assess the similarities to our approach of prioritizing genes according to a lack of variation between diagnoses we analysed our data as suggested when using the ΔCT method.

We also analysed our data using three algorithms (BestKeeper [19], NormFinder [20] and geNorm [21]) designed to rank genes in order of their stability of expression and hence suitability for use as reference genes. These algorithms used different approaches to attempt to identify genes that have expression profiles best suited to them being used as reference genes. Hence, BestKeeper uses repeated pair-wise correlation to identify highly correlated genes, and takes into account the calculated variations of each gene across samples, to choose the best reference genes. The geometric mean of the best reference genes is then calculated to form an index, against which each reference gene can be correlated to describe the relation between the index and the contributing candidate reference gene. NormFinder selects for suitable candidate genes based on minimal variation estimates across all samples, rather than selecting genes with the highest degree of similarity of expression profile across the sample set. This eliminates the possible effects of there being co-regulated genes among the candidates and reduces the risk of rejecting an optimal candidate based on a different pattern of expression across the sample set compared to the other genes being tested. An additional optional step in the analysis allows for identifying the number of genes to include based on the intragroup variance, which was not performed in the current study. Finally, geNorm assesses the variation in expression between two candidate reference genes based on the standard deviation of log-transformed gene ratios. The gene-stability measure *M* is calculated using the average pairwise variation of a gene against all other candidate genes measured. Stepwise exclusion of the gene with the highest *M* value results in decrease of the *M* value until the three most stable genes remain.

For this study we accessed these 3 algorithms using RefFinder [36] which integrates these algorithms and compares the rankings across algorithms; data were entered as mean raw Ct values within each cortical region and all samples and genes were analysed simultaneously.

## Results

### Human CNS collection

There were no significant differences in gender ratio, age, PMI or RINs between subjects with schizophrenia and the controls (Additional file 3: Table S2). There was a small but significant difference in CNS pH with diagnoses, the CNS pH being lower in the tissue from subjects with schizophrenia (mean pH = 6.26) versus controls (mean pH = 6.36; *p* = 0.03).

### Real-time PCR

Regression analyses of data from the standard curves for *GAPDH*, *PPIA*, *SNCA, NOL9*, *TFB1M* and *SKP1* showed the y intercept when x = 0 were -0.004, -0.012, -0.013, - 0.008, -0.018 and 0.0000002, respectively. Thus, whilst these are minor variations they could have major consequences for controlling for the denominator in ongoing analyses of our data and further support the use of non-parametric tests in these analyses.

**Fig. 1:**
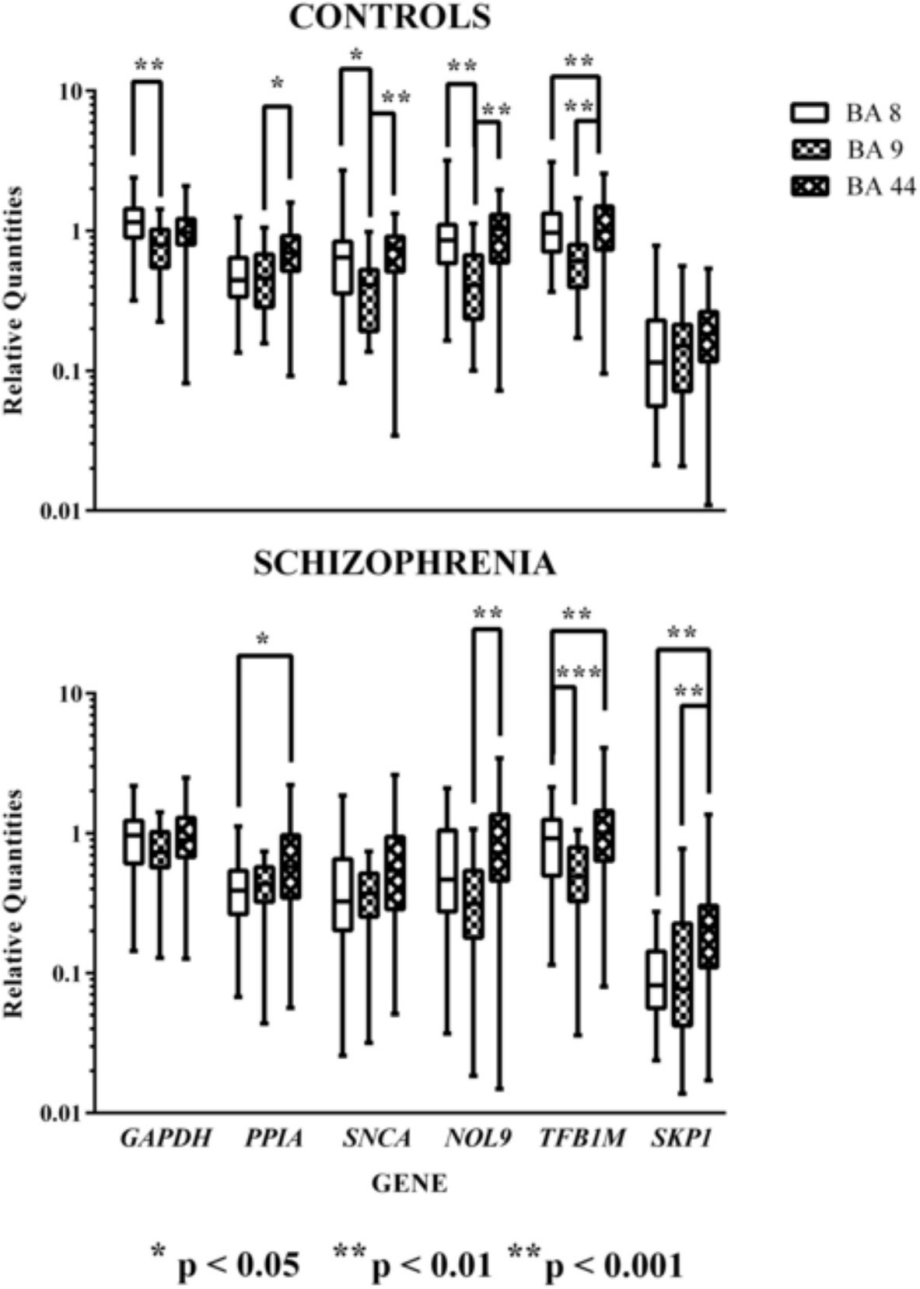
Levels (Median ± Range) of *GAPDH*, *PPIA*, *SNCA*, *NOL9*, *TFB1M* and *SKP1* mRNA in BA 8, 9 and 44 from control subjects and subjects who had schizophrenia

**Fig. 2:**
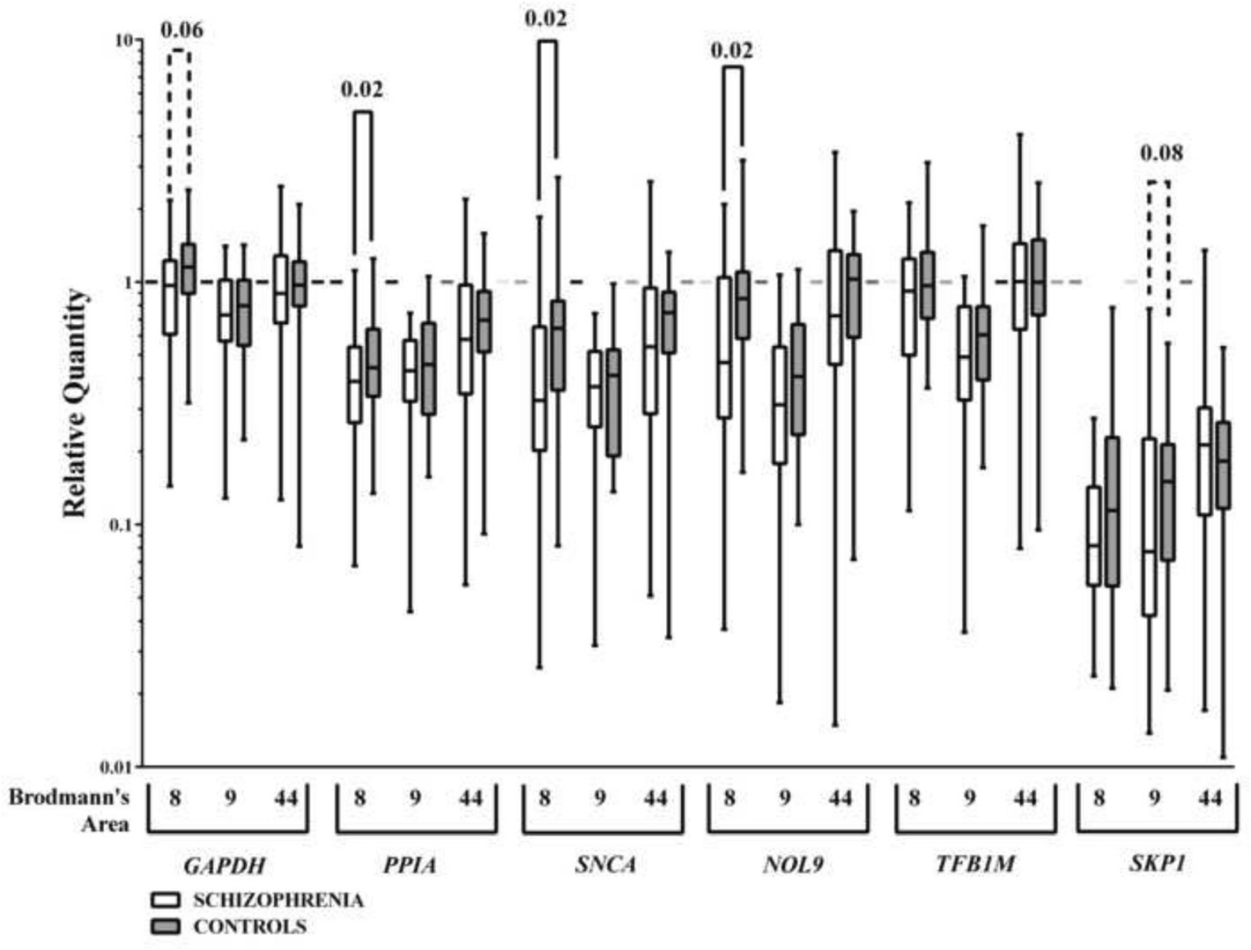
Levels (Median ± Range) of *GAPDH*, *PPIA*, *SNCA*, *NOL9*, *TFB1M* and *SKP1* mRNA in BA 8, 9 and 44 from control subjects and subjects who have had schizophrenia

Using raw data corrected for efficiency of amplification (relative quantities), levels of mRNA for every gene varied between cortical regions in either the control subjects or the subjects with schizophrenia (Fig. 1). Focusing on variation with diagnoses within each cortical region, there were significantly lower levels of *PPIA*, *SNCA* and *NOL9* mRNA as well as a strong trend towards significantly lower levels of *GAPDH* in BA 8 and a trend to lower levels of *SKP1* in BA 9 from subjects with schizophrenia compared to control (Fig. 2).

There are a number of confounds that could be affecting gene expression but our data showed that relationships between levels of *GAPDH*, *PPIA*, *SNCA*, *NOL9* and *TFB1M* mRNA did not show significant deviation from a slope of 0 when correlated with age, PMI, CNS pH and DI in BA 8 (Additional file 4: Table S3). The correlations between RIN and levels of *PPIA* and *TFB1M* as well as SKP1 and pH deviated significantly from 0 but the coefficient of determination showed these relationships to be extremely weak (0.110 to 0.114) (Additional file 5: Figure S2). In BA 9, the correlations between RIN and levels of *GAPDH*, *SNCA*, *NOL9* and *TFB1M* deviated significantly from 0 but the coefficient of determination showed these relationships to be extremely weak (r^2^ from 0.07 to 0.17; Additional file 4: Table S3 and Additional file 5: Figure S2). In BA 44, the correlations between CNS pH and *GAPDH*, *SNCA* and *NOL9* as well as RIN for mRNA levels for all genes deviated significantly from 0 but the coefficient of determination showed these relationships to be extremely weak (r^2^ from 0.078 to 0.163; Additional file 4: Table S3 and Additional file 6: Figure S3). Thus, we concluded that the commonly examined confounds associated with postmortem research in psychiatric disorders were not contributing significantly to the levels of the cortical mRNA measured in this study.

**Fig. 3:**
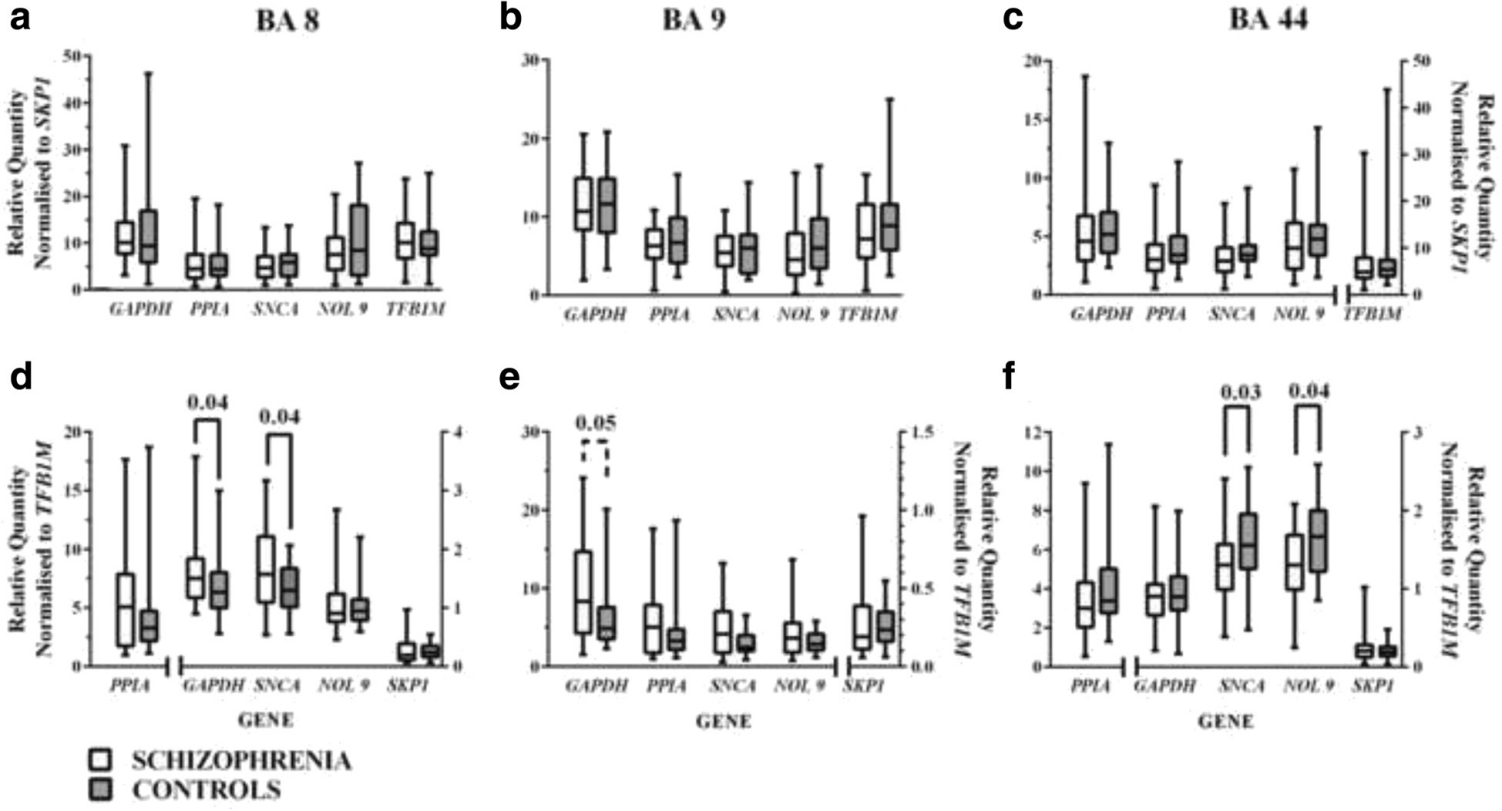
Levels (Median ± Range) of *GAPDH*, *PPIA*, *SNCA*, *NOL9* and *TFB1M* mRNA normalised to *SKP1* mRNA (**a**-**c**) and *GAPDH*, *PPIA*, *SNCA*, *NOL9* and *SKP1* mRNA normalised to *TFB1M* mRNA (**d**-**f**) in BA 8, 9 and 44 from control subjects and subjects who had schizophrenia

To begin assessing the value of using one or more reference genes to normalise data from postmortem CNS we noted that levels of mRNA for *SKP1* and *TFB1M* did not vary significantly with diagnoses in any of the cortical regions studied. Thus, we normalise levels of genes other than the selected reference gene to levels of either *SKP1* or *TFB1M* mRNA. These analyses showed that levels of mRNA of *GAPDH*, *PPIA*, *SNCA*, *NOL9* and *TFB1M * normalised to *SKP1* mRNA did not differ in BA 8, 9 and 44 from subjects with schizophrenia compared to controls (Fig. 3a-c). By contrast, when levels of mRNA were normalised to *TFB1M* levels of *GAPDH* (*p* = 0.04) and *SNCA* (*p* = 0.04) mRNA were significantly higher in BA 8 (Fig. 3d), there was a very strong trend to higher relative expression of *GAPDH* (*p* = 0.05) in BA 9 (Fig. 3e) and the relative expression of *SNCA* (*p* = 0.03) and *NOL9* (*p* = 0.04) were lower in BA 44 (Fig. 3f) in schizophrenia. These data showed the use of a single reference gene runs the risk of introducing a bias and therefore strengthens the argument to use the geometric mean of multiple reference genes.

**Fig. 4:**
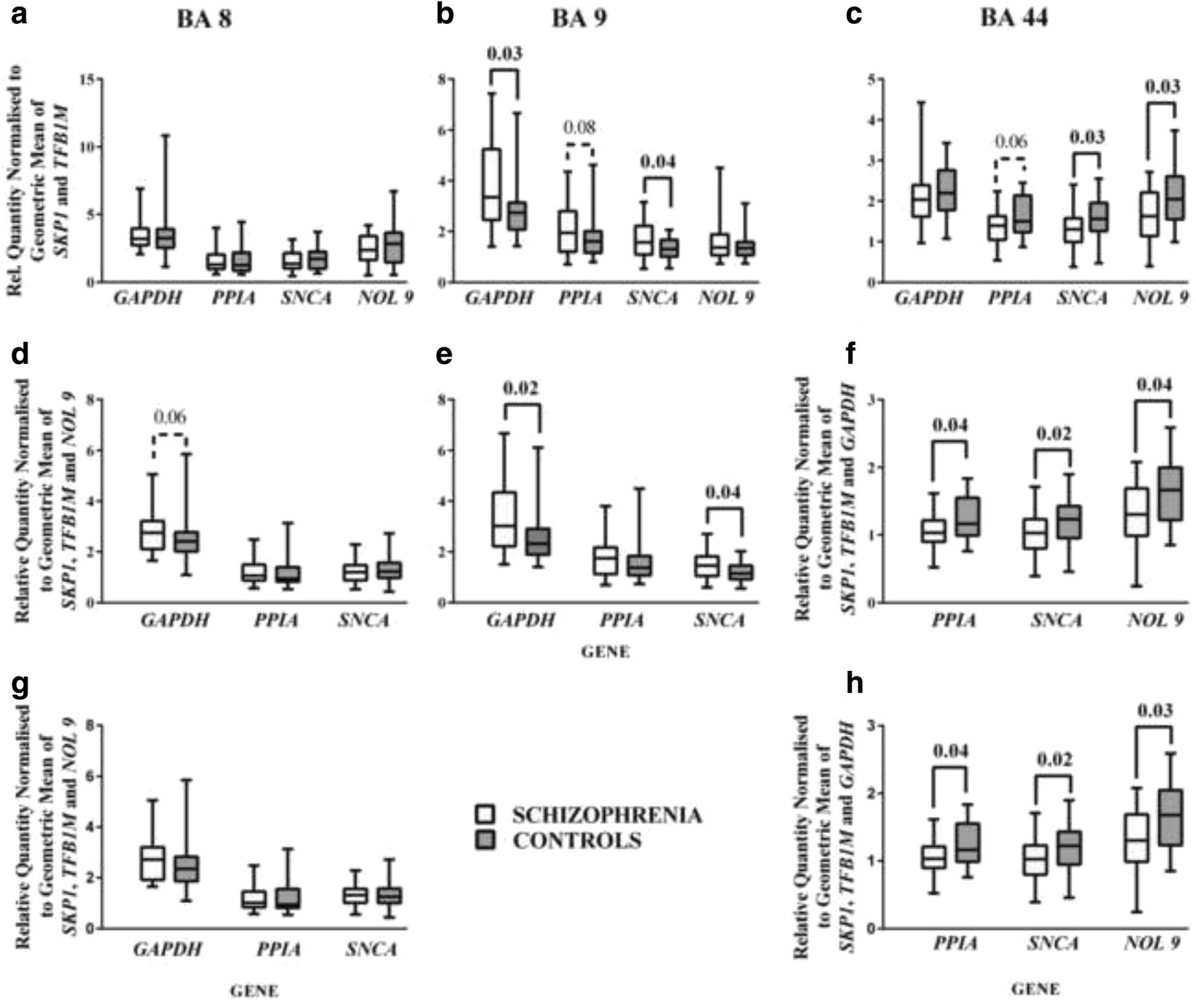
Levels (Median ± Range) of *GAPDH*, *PPIA*, *SNCA*, *NOL9* mRNA normalised to the geometric mean of *SKP1* and *TFB1M* mRNA (**a**-**c**), *GAPDH*, *PPIA*, *SNCA* normalised to the geometric mean of *SKP1*, *TFB1M* and *NOL9* (**d** and **e**) or *PPIA*, *SNCA* and *NOL9* normalised to the geometric mean of *SKP1*, *TFB1M* and *GAPDH* (**f**) mRNA in cortical tissue from control subjects and subjects who have had schizophrenia. Levels (Median ± Range) of *GAPDH*, *PPIA* and *SNCA* normalised to *SKP1*, *TFB1M* and *NOL9* (**g**) or *PPIA*, *SNCA* and *NOL9* normalised to the geometric mean of *SKP1*, *TFB1M* and *GAPDH* (**h**) where only data on mRNA from cases with RIN > 5 is also shown

Next we used the geometric mean of *TFB1M* and *SKP1* to normalise levels of mRNA for the other genes in BA 8, 9 and 44 (Fig. 4a-c). These analyses showed there were no significant differences in levels of mRNA for *GAPDH*, *PPIA*, *SNCA* and *NOL9* in BA 8 (Fig. 4a). In BA 9 there were higher levels of *GAPDH* (*p* = 0.03) and *SNCA* (*p* = 0.04) in the cortex of subjects with schizophrenia (Fig. 4b); there was also a trend (*p* = 0.08) toward higher levels of *PPIA* mRNA. In BA 44 there were lower levels of *SNCA* (*p* = 0.03) and *NOL9* (*p* = 0.03), and a strong trend to lower levels of *PPIA* (*p* = 0.06), mRNA in subjects with schizophrenia (Fig. 4c).

We then used the mean of three reference genes to normalise data, acknowledging that levels of mRNA for all the other genes changed with diagnosis in at least one of the three cortical regions. To overcome this limitation we used the geometric mean of *SKP1*, *TFB1M* and *NOL9* in BA 8 and 9 and the geometric mean of *SKP1*, *TFB1M* and *GAPDH* in BA 44. These analyses showed i) a strong trend to higher levels of *GAPDH* mRNA (*p* = 0.06) in BA 8 (Fig. 4d), ii) higher levels of *GAPDH* (*p* = 0.02) and *SNCA* (*p* = 0.04) mRNA in BA 9 (Fig. 4e) and iii) lower levels of *PPIA* (*p* = 0.04), *SNCA* (*p* = 0.02) and *NOL9* (*p* = 0.04) in BA 44 (Fig. 4f) from subjects with schizophrenia.

### RNA Integrity Number (RIN) as a potential confound in reference gene identification

It has been suggested that qPCR should not be used to measure mRNA in postmortem CNS with RINs < 5 [37]. It was notable in this study that there were no strong correlations between RIN and levels of mRNA in any of the cortical regions studied (Additional file 4: Table S3 and Additional file 6: Figure S3). This suggested that levels of mRNA for the genes of interest in our study were relatively stable compared to the overall measure of mRNA stability provided by RINs. To test this hypothesis we compared levels of mRNA for our genes of interest in BA 8 and BA 44 which had RINs < 5.0 to the data from all cases and showed levels of mRNA did not vary significantly between the two groups (BA 8: *GAPDH p* = 0.66, *PPIA p* = 0.89 and *SNCA p* = 0.41 and BA 44: *GAPDH p* = 0.98, *PPIA p* = 0.95 and *SNCA p* = 0.96). We then compared levels of mRNA across diagnoses in BA 8 and BA 44 excluding data from cases with RIN < 5; analyses which showed the loss of the strong trend to higher levels of GAPDH in BA 8 from subjects with schizophrenia (Fig. 4g) that was present using data from the whole cohort (Fig. 4d). By contrast, the lower levels of mRNA for *PPIA*, *SNCA* and *NOL9* that were apparent in BA 44 when all the data was included in the analyses (Fig. 4f) were still significantly lower (*PPIA* (*p* = 0.04), *SNCA* (*p* = 0.02) and *NOL 9* (*p* = 0.03) when only case with a RIN > 5.0 were included (Fig. 4h).

### Comparison of different reference gene identification methodologies

**Fig. 5:**
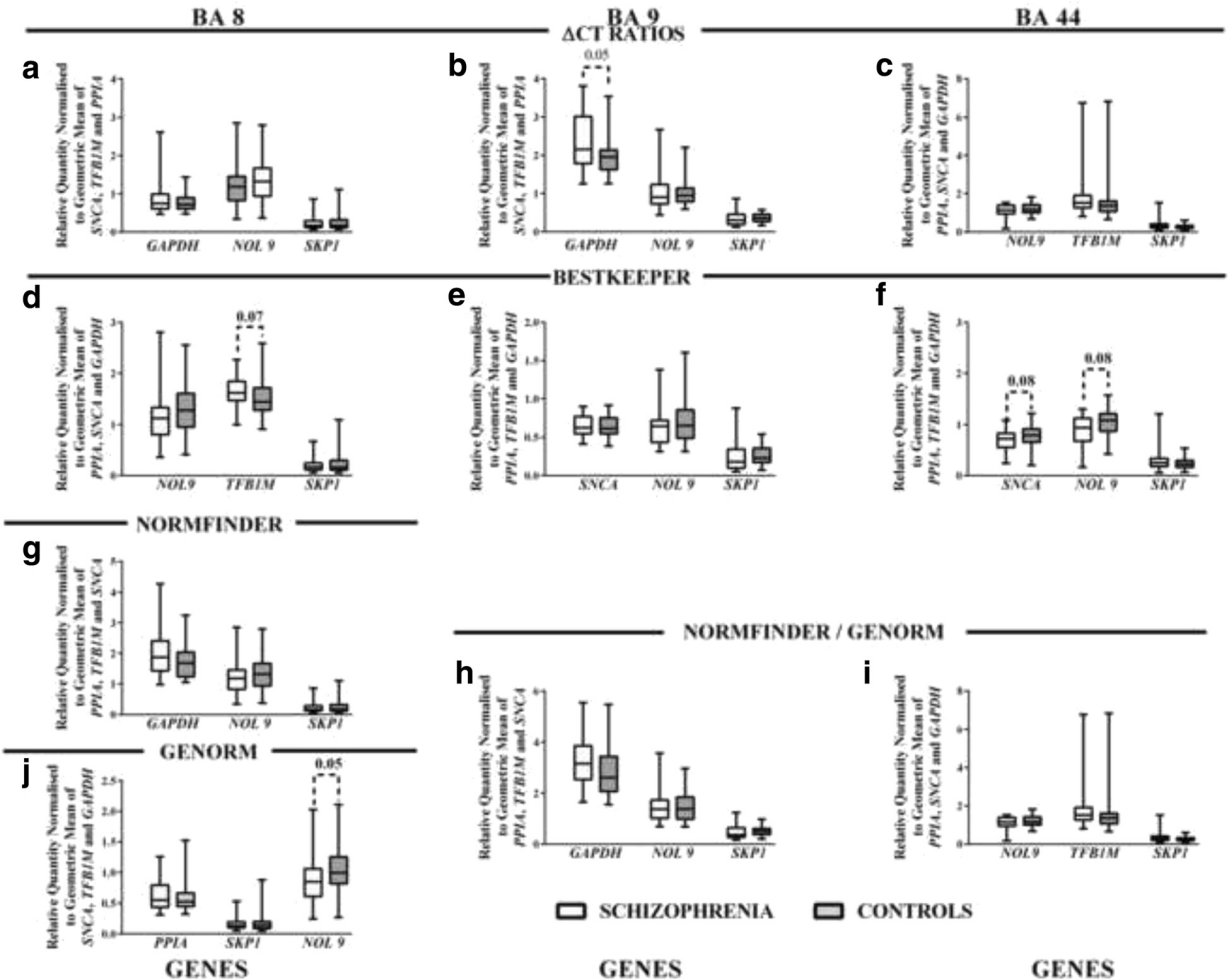
Levels (Median ± Range) of genes of interest in BA 8, 9 and BA 44 normalised to the geometric mean of reference genes identified using ΔCT Ratios (**a**-**c**), BestKeeper (**d**-**f**), NormFinder (**g**-**i**) or geNorm (**j**,**h**,**i**) ranking

When our ΔCT ratios underwent parametric analyses, as is suggested when using this model, the best performing genes (ranked according to the mean of the standard deviations for each gene pair ratio) in BA 8 were *SNCA*, *TFB1M* and *PPIA*, in 9 were *SNCA, PPIA* and *TFB1M* and in BA 44 were *PPIA*, *GAPDH* and *SNCA* (Additional file 7: Table S4). We then compared data from the 3 genes not used as reference genes and expressed as the geometric mean of *SNCA*, *TFB1M* and *PPIA* in BA 8, *SNCA, PPIA* and *TFB1M* in BA 9 and *PPIA*, *GAPDH* and *SNCA* in BA 44 from subjects with schizophrenia and controls. There were no significant changes in mRNA levels between diagnoses in BA 8 (Fig. 5a) or 44 (Fig. 5c); there was a strong trend to higher levels of GAPDH mRNA in BA 9 from subjects with schizophrenia (Fig. 5b).

A number of algorithms have been developed that can be used to rank genes in order of stability for use as reference genes. In BA 8, no algorithms rank the same three genes as the best candidates to use as reference genes (Additional file 8: Table S5). Using the suggested 3 genes from BestKeeper showed a trend to higher levels of *TFB1M* in BA 8 from subjects with schizophrenia (Fig. 5d), NormFinder did not reveal any significant variation with diagnoses in that cortical region (Fig. 5g) whilst using reference genes suggested by geNorm showed a strong trend to lower levels of *NOL9* in subjects with schizophrenia (Fig. 5j). BestKeeper suggested the same 3 genes (*GAPDH, TFB1M* and *PPIA*) should be used as reference genes in BA 9 and 44; using these reference genes meant there was no significant variation in levels of mRNA with diagnoses in BA 9 (Fig. 5e) but there were trends to lower levels of *SNCA* and *NOL9* in BA 44 for people with schizophrenia (Fig. 5f). NormFinder and geNorm gave the same sets of three genes as the best to use as reference genes in BA 9 (*SNCA*, *PPIA* and *TFB1M*) and BA 44 (*PPIA*, *GAPDH* and *SNCA*). Using the appropriate sets of reference genes showed there were no significant variations in levels of mRNA with diagnoses in BA 9 of BA 44 (Fig. 5h and i) in schizophrenia compared to controls.

## Discussion

Identifying reference genes that truly fulfil the required criteria for such genes [3] has been shown to be difficult. Hence, we took our data quantifying mRNA in three cortical regions from subjects with schizophrenia and controls and subjected them to analyses using a structured “in house” statistically driven approach that initially involved using minimally derived data and compared the three genes that appeared to have the best properties associated with reference genes to those recommended by four other approaches to identifying reference genes using qPCR data. Of the 6 genes we measured that could have potentially fulfilled the criteria to be reference genes, we found that levels of mRNA for all of the genes varied across cortical regions; these data add to that of others [4–6] to argue that finding genes with stable expression across many CNS regions will be extremely difficult. One study has reported levels of mRNA for G protein pathway suppressor 1 (*GPS1*) and ubiquitin conjugating enzyme E2D 2 (*UBE2D2*) and suggested these genes could be used as CNS wide reference genes [38]. However, that study was carried out using tissue from two donors and therefore lacked significant power to discover inter-person variability in gene expression across the CNS but the study provides preliminary data to support the notion that some genes might be able to be used as reference genes across CNS regions. Thus, until these data are replicated in larger collections of CNS it would seem that comparisons of gene expression in post-mortem CNS across diagnoses may need to be carried out within a CNS region without comparing results across multiple CNS regions.

As we could not use a single set of reference genes across multiple cortical regions to normalise qPCR data we then determined if any of our six genes could be used as reference genes within a cortical region. Using our statistically driven approach we identified up to three of the six genes which fulfilled the criteria for reference genes within each cortical region, that is levels of mRNA did not vary with diagnoses in that region. Then, within each cortical region, we confirmed that using multiple reference genes gave more stable outcomes although we did not observe any great differences in outcomes using two, compared to the recommended three [13], reference genes. Our data also suggested that if levels of mRNA are not correlated to RIN then data from tissue where cases had RINs < 5.0 did not affect comparisons of data across diagnoses. Finally, we showed that the use of four different approaches to identifying reference genes did not suggest the same genes which obviously affected subsequent comparisons across diagnoses within each of the three cortical regions. Thus, our study emphasises that the selection of reference genes is critical for studies examining gene expression using postmortem CNS from subjects with psychiatric disorders such as schizophrenia, and this must be acknowledged as a confound when comparing results from different studies.

Turning to our data across diagnoses, our analyses using the geometric mean of levels of mRNA of three region specific reference genes identified using our internal approach showed that levels of *GAPDH* and *SNCA* mRNA were higher in BA 9 from subjects with schizophrenia compared to controls. By contrast, in BA 44 levels of *PPIA*, *SNCA* and *NOL9* were lower in subjects with schizophrenia. Our data are partly supported by another study that reported higher levels of *GAPDH* mRNA in the cortex of subjects with schizophrenia [39]. Notably, GAPDH protein has been reported as decreased in the cortex from subjects with schizophrenia [40], suggesting the increase in gene expression may be an attempt to compensate for low protein levels. As in our study, lower levels of *PPIA* mRNA have been reported in the cortex of subjects with schizophrenia [41]. By contrast we are aware of no studies reporting change in levels of *SNCA* or *NOL9* mRNA in the cortex of subjects with schizophrenia. However, the presence of higher levels of SNCA protein in the layer 2 of the insular cortex suggests changes in *SNCA* gene expression are present in the CNS of subjects with schizophrenia [42].

*GAPDH* encodes a protein that has roles in glycolysis and the control of nuclear function and so it can influence many aspects of cellular development [43], *SNCA* encodes a protein that is a pre-synaptic protein involved in neurotransmitter release [44], *PPIA* encodes a protein that is an intracellular protein that regulates protein folding and trafficking [45] whilst *NOL9* encodes a protein that is a novel polynucleotide 5′-kinase involved in ribosomal RNA processing [46]. Hence changes in the expression of any of these four genes could have profound effects on cortical function and therefore deserve further investigation, but such investigations were beyond the scope of our current studies.

Analysing our data on mRNA levels for six genes from 60 individuals in three cortical regions from subjects with schizophrenia shows the choice of reference genes can have a significant impact on the outcomes from subsequent statistical analyses. Moreover, whilst the notion of expressing results as a ratio to a reference gene to attempt to control for methodological variables such as RNA extraction and cDNA synthesis has clear merit, the subsequent need for statistical analyses using ratios is a clear confound. There is a growing focus on making sure researchers impose high levels of quality control on their methodologies [47]; this is particularly important when completing a quantitative methodology. Whilst the conflict between the need for the use of reference genes and the subsequent problems in data analyses seems unresolvable there are some ways to minimise the impact of potential ambiguity in analysing data in the form of ratios. First, linear regression analyses of the standard curve for each gene should be completed and the y-axis intercept determined. If the y-axis intercept for these lines is not zero and does vary between gene then the subsequent data analyses should use non-parametric approaches [15]. The analysis of our data would argue that, when comparing levels of mRNA in the same CNS region from subjects with different psychiatric disorders, using minimally derived data as a starting point to identifying potential reference genes is worthwhile as it can show differences of level of absolute gene expression with diagnoses. We would argue that, whether or not these differences are real or are reflective of some bias in RNA processing, the use of such genes as reference genes should be avoided. The suggestion that all data on genes of interest should be expressed as the geometric mean of three reference gene to stabilise outcomes from subsequent statistical analyses [13] has clear merit. However it could be worthwhile to present analyses of qPCR data at the level of underived data and as a geometric mean as one method may show differences because of sample processing bias and the other of statistical processing but, more importantly, if both analyses show the same outcome then this would suggest any differences are due to disease process and not sample or data processing.

## Conclusions

Our attempts to identify suitable reference genes to investigate changes in gene expression in multiple regions of the cortex from subjects with schizophrenia and controls has added to the notion that reference genes, according to the definitive criteria, do not exist in the human CNS. Our data suggests comparing levels of gene expression normalised to multiple reference genes are possible within defined cytoarchitectural regions which may mean it could be necessary to identify specific reference genes for discrete nuclei such as those in the hippocampus and amygdala. Our data argues that all studies reporting qPCR data using normalised gene expression need to show, using minimally derived data, that levels of mRNA for the reference genes do not vary with diagnoses or conditions, as set out in the MIQE guidelines [48], rather than simply stating the genes used were picked using ranking algorithms. In addition, as we and others [11], have shown, the use of algorithms to identify reference genes is a confound within any study that should be acknowledged. We have also recommended other analyses that should be completed and reported to give added comfort that an effort has been made to address the conflict between the need to express qPCR data as a ratio of reference genes and subsequent analyses of such derived data. Finally, as has been suggested previously [49], the problem in finding reference genes in the human CNS should be noted when measuring proteins using Western blots where supposed reference proteins (loading controls), are used to normalise data even though some proteins used as loading controls, such as GAPDH, have been shown to vary in levels in the CNS of subjects with psychiatric disorders.

### Ethics and consent to participate

Approval to collect tissue used in these studies was obtained from the Ethics Committee of the Victorian Institute of Forensic Medicine and the Mental Health Research Institute (VIFM EC 3/2–13). Tissue was collect after approval was obtained from the next of kin.

### Consent to publish

This manuscript contains data on gender, duration of illness and postmortem interval from each donor. Permission to publish such information was gained as part of the consent to collect tissue.

### Availability of data and materials

Experimental data from this study is owned by the CRC for Mental Health but could be made available on request. All non-experimental data is provided accept age. Information on age will not be provided to ensure anonymity of each tissue donor.

## Additional files

Additional file 1: Table S1. Primer sequences and accession numbers for the primers used in qPCR. (DOCX 15 kb) Additional file 2: Figure S1. Examples of plate wide melt curves for *GAPDH*, *PPIA*, *SNCA*, *NOL 9*, *TFB1M* and *SKP 1*. (DOCX 3905 kb) Additional file 3: Table S2. Demographic, medical, treatment and CNS collection data for the cases from which tissue was obtained for this study. Individual ages are not given to ensure donor anonymity. (DOCX 29 kb) Additional file 4: Table S3. The relationships between levels of cortical mRNA and donor age, post-mortem interval (PMI), CNS pH, duration of illness (DI) and RNA integrity number (RIN). Relationships where the regression line deviated significantly from a slope of zero are bolded and in italics. (DOCX 24 kb) Additional file 5: Figure S2. The relationship between levels of mRNA and RNA Integrity Number (RIN) for *PPIA* (A) and *TFB1M* (B) as well as CNS pH and *SKP1* (C) in BA 8 as well as *GAPDH* (D), *SNCA* (E), *TFB1M* (F) and *NOL 9* (G) with RIN in BA 9. The linear regression line is shown ± 95 % prediction confidences. (DOCX 102 kb) Additional file 6: Figure S3. The relationship between levels of mRNA and CNS pH for *GAPDH* (A), *SNCA* (B) and *NOL9* (C) as well as RNA Integrity Number (RIN) and *GAPDH* (D), *PPIA* (E), *SNCA* (F), *NOL 9* (G), *TFB1M* (H) and *SKP1* (I) in BA 44. The linear regression line is shown ± 95 % prediction confidences. (DOCX 131 kb) Additional file 7: Table S4. A comparison of the mean standard deviation (Mean STD) for each potential reference gene expressed as a ratio of mRNA for one gene to all remaining genes in the three regions of the human cortex. STD = standard deviation of data for each individual gene. (DOCX 20 kb) Additional file 8: Table S5. Genes suggested as most suitable to use as reference genes by three different algorithms. (DOCX 18 kb)

## Abbreviations

BA: Brodmann’s area
DI: duration of illness
FRADD: final recorded antipsychotic drug dose in mg chlorpromazine equivalents per day
GAPDH: glyceraldehyde-3-phosphate Dehydrogenase
LEAP: lifetime exposure to such drugs
NOL9: nucleolar protein 9
PPIA: peptidylprolyl isomerase A
qPCR: quantitative polymerase chain reaction
RINs: RNA integrity numbers
SKP1: S-phase kinase-associated protein 1
SNCA: alpha-synuclein
TFB1M: transcription factor B1, Mitochondrial
