# Supplementary material for: Validating reference genes using minimally transformed qpcr data: findings in human cortex and outcomes in schizophrenia
Source: BMC Psychiatry. 2016 May 20;16:154. doi: 10.1186/s12888-016-0855-0 (PMC4875643; doi:10.1186/s12888-016-0855-0)
Supplement: Additional file 1: Table S1. — Primer sequences and accession numbers for the primers used in qPCR. (DOCX 15 kb) [file 12888_2016_855_MOESM1_ESM.docx]

|  | primer sequences | mRNA accession numbers |
| --- | --- | --- |
| *NOL9* | F ccactttctcctttacatagc | NM_024654.4 |
|  | R acaattcacggtccttagc |  |
| *SKP1* | F cctcctcctcctgaagatg | variant 1 NM_006930.3 |
|  | R aaactgtgtgctacctacc |  |
| *TFB1M* | F cgtagtcgcctctctgttatgg | NM_016020.3 |
|  | R gctgctctatcttgggctgtatc |  |
| *PPIA* | F atggtcaaccccaccgtgttcttcg | variant 1 NM_021130.4 |
|  | R cgtgtgaagtcaccaccctgacaca |  |
| *SNCA* | F ctgctgctgagaaaAccaaa | variants 1-4 NM_000345.3,  NM_007308.2 |
|  | R ctgctccctccactgtctt |  |
| *GAPDH* | F tgcaccaccaactgcttagc | variants 1-4 NM_002046.5, NM_001256799.2 |
|  | R ggcatggactgtggtcatgag |  |

Supplementary Table 1: Primer sequences and accession numbers for the primers used in qPCR.
