# Supplementary figures and images for: Validating reference genes using minimally transformed qpcr data: findings in human cortex and outcomes in schizophrenia
Source: BMC Psychiatry. 2016 May 20;16:154. doi: 10.1186/s12888-016-0855-0 (PMC4875643; doi:10.1186/s12888-016-0855-0)

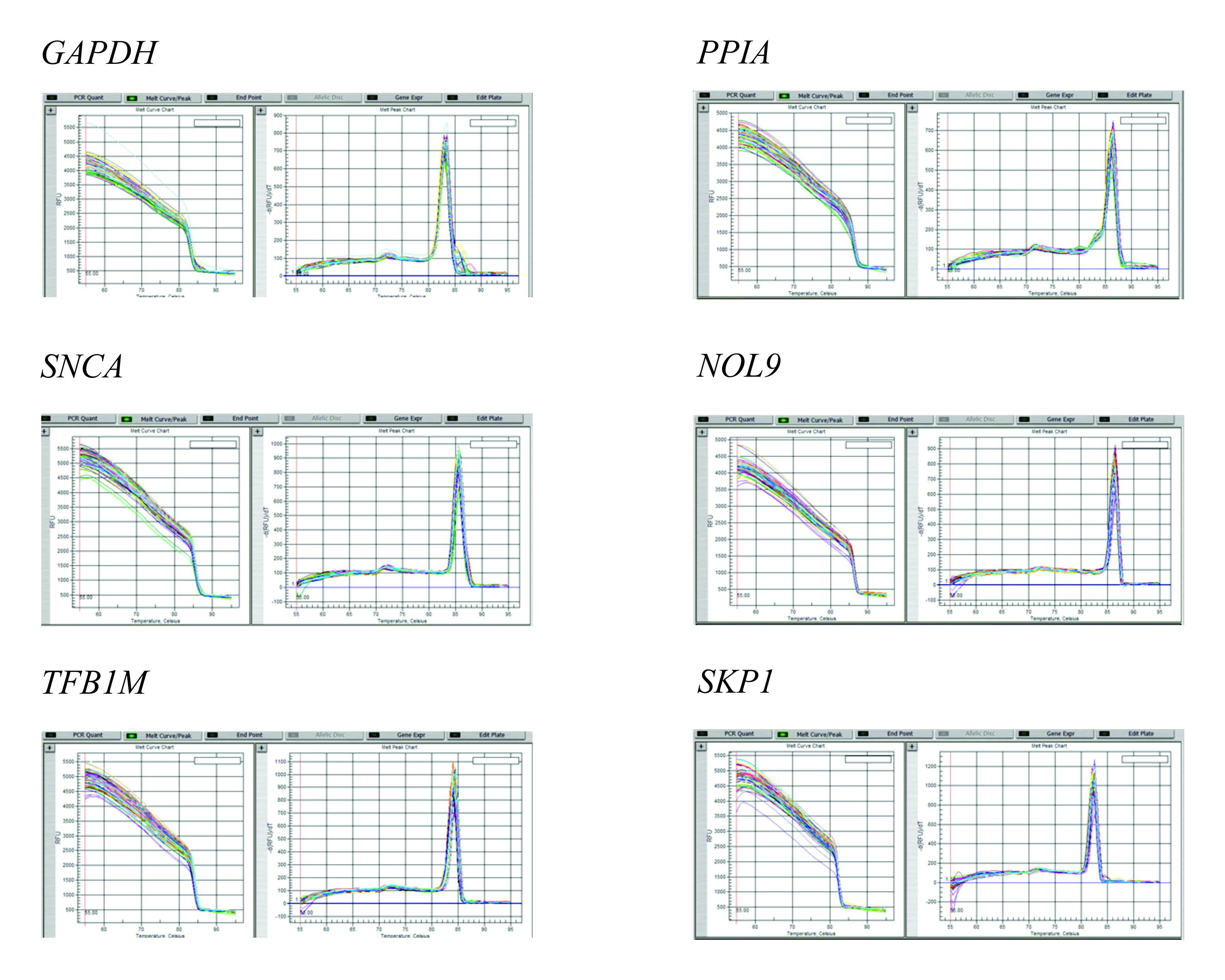


Supplementary Figure 1: Examples of plate wide melt curves for *GAPDH*, *PPIA*, *SNCA*, *NOL 9*, *TFB1M* and *SKP 1*.

Supplement: Additional file 2: Figure S1. — Examples of plate wide melt curves for GAPDH, PPIA, SNCA, NOL 9, TFB1M and SKP 1. (DOCX 3905 kb) [file 12888_2016_855_MOESM2_ESM.docx]
