# Supplementary material for: Validating reference genes using minimally transformed qpcr data: findings in human cortex and outcomes in schizophrenia
Source: BMC Psychiatry. 2016 May 20;16:154. doi: 10.1186/s12888-016-0855-0 (PMC4875643; doi:10.1186/s12888-016-0855-0)
Supplement: Additional file 3: Table S2. — Demographic, medical, treatment and CNS collection data for the cases from which tissue was obtained for this study. Individual ages are not given to ensure donor anonymity. (DOCX 29 kb) [file 12888_2016_855_MOESM3_ESM.docx]

Supplementary Table 2: Demographic, medical, treatment and CNS collection data for the cases from which tissue was obtained for this study. Individual ages are not given to ensure donor anonymity.

| **SCHIZOPHRENIA** | | | | |  |  |  |  |  |  | **RIN** |  |  |  |  |
| --- | --- | --- | --- | --- | --- | --- | --- | --- | --- | --- | --- | --- | --- | --- | --- |
|  | **Sex** | | **Age** | | **Sui** | **PMI** | **DI** | **Cause of Death** | **pH** | **BA 8** | **BA 9** | **BA 44** | **Antipsychotic Drugs** | **FRADD** | **LEAP** |
|  |  | | **(yr.)** | |  | **(hr.)** | **(yr.)** |  |  |  |  |  |  |  |  |
|  | F | |  | | N | 36 | 48 | Chronic Obstructive Airways Disease | 5.84 | 8.0 | 8.2 | 7.3 |  |  |  |
|  | M | |  | | N | 39.5 | 45 | Bronchopneumonia | 6.49 | 7.4 | 8.8 | 8.6 | Chlorpromazine | 1200 | 54.000 |
|  | M | |  | | N | 20 | 32 | Ischaemic Heart Disease | 5.98 | 4.6 | 9.5 | 8.4 | Fluphenazine Thioridazine | 2000 | 64.000 |
|  | M | |  | | N | 32.5 | 27 | Ischaemic Heart Disease | 6.41 | 6.9 | 9.1 | 7.8 | Fluphenazine Thioridazine | 530 | 14.310 |
|  | M | |  | | Y | 22 | 8 | Burning | 6.28 | 4.3 | 8.6 | 7.9 | Chlorpromazine Pimozide | 1200 | 9.600 |
|  | F | |  | | N | 58.5 | 37 | Aspiration: Pneumonia | 6.48 | 7.1 | 8.8 | 7 | Chlorpromazine | 25 | 0.925 |
|  | M | |  | | Y | 49 | 2 | Drug Overdose | 6.38 | 6.1 | 9.3 | 8.2 | Trifluoperazine | 200 | 0.400 |
|  | M | |  | | N | 37 | 30 | Intestinal Ischemia | 5.98 | 6.1 | 9.3 | 5.9 | Fluphenazine Chlorpromazine | 1700 | 51.000 |
|  | M | |  | | N | 21 | 36 | Pneumonia | 6.46 | 6.9 | 9.1 | 7.7 | Fluphenazine | 75 | 2.700 |
|  | M | |  | | N | 32 | 23 | Ischaemic Heart Disease | 6.28 | 7.4 | 9.1 | 8.6 | Thioridazine | 600 | 13.800 |
|  | M | |  | | Y | 41.5 | 21 | Multiple Injuries | 6.52 | 6.5 | 9.4 | 8.6 | Chlorpromazine Haloperidol | 1400 | 29.400 |
|  | M | |  | | N | 37 | 3 | Pericarditis | 6.07 | 7.5 | 9 | 7.9 | Trifluoperazine Flupenthixol | 450 | 1.350 |
|  | M | |  | | N | 48 | 53 | Aspiration: Food | 6.45 | 4.2 | 8.6 | 5.8 | Thioridazine | 150 | 7.950 |
|  | M | |  | | N | 43 | 7 | Aspiration: Food | 6.23 | 4.3 | 9.3 | 8.5 |  |  |  |
|  | M | |  | | N | 44.5 | 47 | Ischaemic Heart Disease | 6.38 | 4.7 | 8.2 | 7.9 | Trifluoperazine | 100 | 4.700 |
|  | M | |  | | Y | 37 | 3 | Combined Drug Toxicity | 6.17 | 2.6 | 9.6 | 8.4 | Pimozide | 200 | 0.600 |
|  | M | |  | | N | 50 | 4 | Meningo Encephalitis | 6.02 | 2.9 | 8.8 | 6.8 | Chlorpromazine Haloperidol Clozapine | 100 | 0.400 |
|  | F | |  | | N | 15 | 7 | Coronary Artery Thrombosis | 6.26 | 4.3 | 9 | 7.8 | Haloperidol | 300 | 2.100 |
|  | M | |  | | N | 25 | 33 | Coronary Artery Atheroma | 6.1 | 4.3 | 9.3 | 5.3 | Thioridazine | 400 | 13.200 |
|  | F | |  | | N | 52.5 | 22 | Pulmonary Thromboembolism | 6.21 | 7.3 | 8.8 | 4.1 | Fluphenazine Chlorpromazine | 700 | 15.400 |
|  | M | |  | | N | 42 | 36 | Bronchopneumonia | 6.29 | 6.2 | 8.5 | 8.7 | Trifluoperazine Haloperidol | 460 | 16.560 |
|  | F | |  | | N | 50 | 18 | Ruptured Abdominal Aneurysm | 6.35 | 7.2 | 8.3 | 7.9 | Fluphenazine Haloperidol | 550 | 9.900 |
|  | M | |  | | Y | 47 | 8 | Hanging | 6.44 | 5.5 | 8.4 | 7.4 | Haloperidol | 128 | 1.024 |
|  | M | |  | | Y | 31 | 11 | Combined Drug Toxicity | 6.20 | 5.5 | 8.6 | 7.7 | Fluphenazine Trifluoperazine | 500 | 5.500 |
|  | M | |  | | Y | 78 | 5 | Multiple Injuries | 6.19 | 7.6 | 9.6 | 8.6 | Haloperidol | 300 | 1.500 |
|  | M | |  | | N | 47 | 22 | Coronary Artery Atheroma | 6.26 | 5.1 | 7.5 | 8.2 |  |  |  |
|  | M | |  | | Y | 52 | 2 | Carbon Monoxide Poisoning | 6.39 | 7.5 | 7.7 | 7.8 | Haloperidol | 500 | 1.000 |
|  | M | |  | | N | 46 | 20 | Bronchopneumonia | 5.80 | 7.3 | 8.4 | 6.8 |  |  |  |
|  | F | |  | | N | 50 | 20 | Pneumonia | 6.31 | 8.4 | 8.8 | 5.5 | Risperidone | 600 | 12.000 |
|  | M | |  | | N | 30 | 24 | Bronchopneumonia | 6.62 | 7.5 | 8.9 | 8.2 | Flupenthixol | 1250 | 30.000 |
| Mean |  | | 49 | |  | 40 | 22 |  | 6.26 | 6.04 | 8.82 | 7.51 |  | 600 | 14 |
| SEM |  | | 2.97 | |  | 2.39 | 2.81 |  | 0.04 | 0.29 | 0.1 | 0.21 |  | 105 | 3.5 |
|  | |  | |  |  |  |  |  |  |  |  |  |  |  |  |
| **CONTROLS** | | | |  |  |  |  |  |  |  |  |  |  |  |  |
|  | M | |  | | N | 50 |  | Ischaemic Heart Disease | 6.33 | 6.5 | 10 | 7.1 |  |  |  |
|  | F | |  | | N | 20.5 |  | Congestive Cardiac failure | 6.58 | 6.8 | 9.8 | 7.8 |  |  |  |
|  | M | |  | | N | 41 |  | Ischaemic Heart Disease | 6.56 | 5.3 | 9.8 | 6.9 |  |  |  |
|  | F | |  | | N | 28 |  | Cancer Uterus | 6.37 | 6.1 | 9.2 | 4.2 |  |  |  |
|  | F | |  | | N | 38 |  | Acute Asthma | 6.32 | 7.6 | 7.4 | 8.6 |  |  |  |
|  | M | |  | | N | 69 |  | Ischaemic Heart Disease | 6.43 | 6.6 | 8.9 | 7.7 |  |  |  |
|  | M | |  | | N | 20.5 |  | Acute Myocardial Infarct | 6.47 | 7.4 | 8.5 | 8.4 |  |  |  |
|  | M | |  | | N | 65 |  | Ischaemic Heart Disease | 6.4 | 7.7 | 8.8 | 8.9 |  |  |  |
|  | M | |  | | N | 50 |  | Exsanguination | 6.48 | 7 | 7.6 | 8 |  |  |  |
|  | M | |  | | N | 44.5 |  | Ischaemic Heart Disease | 6.56 | 7.3 | 9 | 8.7 |  |  |  |
|  | M | |  | | N | 51 |  | Exsanguination | 6.58 | 4.5 | 8.6 | 8.4 |  |  |  |
|  | F | |  | | N | 60 |  | Dilated Cardiomyopathy | 6.4 | 3.3 | 10 | 5.7 |  |  |  |
|  | M | |  | | N | 46.5 |  | Acute Myocardial Infarct | 6.08 | 5.1 | 9.6 | 8.4 |  |  |  |
|  | M | |  | | N | 62 |  | Iatrogenic Haemorrhage | 6.39 | 4.8 | 9.5 | 6 |  |  |  |
|  | M | |  | | N | 35 |  | Right Ventricular Hypertrophy | 6.15 | 6.3 | 9.7 | 7.9 |  |  |  |
|  | M | |  | | N | 46.5 |  | Electrocution | 6.37 | 6.9 | 9.5 | 7.1 |  |  |  |
|  | M | |  | | N | 63 |  | Cardiomegaly | 6.34 | 3.4 | 8.4 | 8.8 |  |  |  |
|  | F | |  | | N | 43 |  | Acute Myocardial Infarct | 6.37 | 4.5 | 9 | 8.4 |  |  |  |
|  | M | |  | | N | 45 |  | Drowning | 6.25 | 6.7 | 9.8 | 7.9 |  |  |  |
|  | M | |  | | N | 24 |  | Electrocution | 6.42 | 7.4 | 9.3 | 6.1 |  |  |  |
|  | M | |  | | N | 24 |  | Coronary Artery Atheroma | 6.37 | 5.2 | 9.7 | 6.3 |  |  |  |
|  | M | |  | | N | 39 |  | Coronary Artery Atheroma | 6.21 | 5.3 | 8.5 | 7 |  |  |  |
|  | M | |  | | N | 56 |  | Ischaemic Heart Disease | 6.38 | 5.8 | 8.9 | 7.6 |  |  |  |
|  | M | |  | | N | 41 |  | Aortic Stenosis | 6.06 | 5.7 | 9.3 |  |  |  |  |
|  | M | |  | | N | 69 |  | Coronary Artery Atheroma | 6.59 | 6.2 | 8.6 | 7.8 |  |  |  |
|  | M | |  | | N | 22 |  | Pulmonary Thromboembolism | 5.98 | 6.3 | 8.6 |  |  |  |  |
|  | M | |  | | N | 51 |  | Coronary Artery Atheroma | 6.43 | 6.9 | 9.1 | 8.9 |  |  |  |
|  | M | |  | | N | 12 |  | Pulmonary Thromboembolism | 6.34 | 5.4 | 7 | 8.5 |  |  |  |
|  | F | |  | | N | 65 |  | Mitral Valve Prolapse | 6.38 | 7 | 7.5 | 7.9 |  |  |  |
|  | M | |  | | N | 26 |  | Coronary Artery Atheroma | 6.32 | 5.1 | 8.3 | 7.4 |  |  |  |
| Mean |  | | 49 | |  | 44 |  |  | 6.36 | 6.00 | 8.93 | 7.59 |  |  |  |
| SEM |  | | 3.00 | |  | 2.95 |  |  | 0.03 | 0.22 | 0.15 | 0.21 |  |  |  |
| p | 1.00 | | | 0.99 |  | 0.41 |  |  | **0.03** | 0.92 | 0.52 | 0.80 |  |  |  |

Abbreviations: BA = Brodmann’s area, Di = duration of illness, FRADD = final recorded antipsychotic drug dose expressed as chlorpromazine equivalents, LEAP = lifetime exposure to antipsychotic drugs expressed as drugs in chlorpromazine equivalents * 10^-3^, PMI = postmortem interval, RIN = RNA integrity number, Sui = suicide.
