# Supplementary material for: Validating reference genes using minimally transformed qpcr data: findings in human cortex and outcomes in schizophrenia
Source: BMC Psychiatry. 2016 May 20;16:154. doi: 10.1186/s12888-016-0855-0 (PMC4875643; doi:10.1186/s12888-016-0855-0)
Supplement: Additional file 4: Table S3. — The relationships between levels of cortical mRNA and donor age, post-mortem interval (PMI), CNS pH, duration of illness (DI) and RNA integrity number (RIN). Relationships where the regression line deviated significantly from a slope of zero are bolded and in italics. (DOCX 24 kb) [file 12888_2016_855_MOESM4_ESM.docx]

Supplementary Table 3: The relationships between levels of cortical mRNA and donor age, post-mortem interval (PMI), CNS pH, duration of illness (DI) and RNA integrity number (RIN). Relationships where the regression line deviated significantly from a slope of zero are bolded and in italics.

| Cortical Region |  | *GAPDH* | | *PPIA* | | *SNCA* | | *NOL9* | | *TFB1M* | | *SKP1* | |
| --- | --- | --- | --- | --- | --- | --- | --- | --- | --- | --- | --- | --- | --- |
|  |  | r^2^ | p | r^2^ | p | r^2^ | p | r^2^ | p | r^2^ | p | r^2^ | p |
| BA 8 | Age | 0.049 | 0.09 | 0.028 | 0.20 | 0.047 | 0.10 | 0.057 | 0.07 | 0.039 | 0.13 | 0.003 | 0.66 |
|  | PMI | 0.003 | 0.69 | 0.010 | 0.44 | <0.001 | 0.95 | <0.001 | 0.93 | 0.001 | 0.85 | 0.021 | 0.27 |
|  | pH | 0.013 | 0.38 | 0.047 | 0.10 | 0.022 | 0.26 | 0.009 | 0.48 | 0.041 | 0.12 | ***0.110*** | ***0.01*** |
|  | DI | 0.009 | 0.61 | 0.008 | 0.63 | 0.021 | 0.45 | 0.003 | 0.79 | 0.013 | 0.55 | 0.050 | 0.24 |
|  | RIN | 0.058 | 0.06 | ***0.113*** | ***0.01*** | 0.037 | 0.14 | 0.019 | 0.30 | ***0.114*** | ***0.01*** | 0.021 | 0.27 |
|  |  |  |  |  |  |  |  |  |  |  |  |  |  |
| BA 9 | Age | 0.012 | 0.41 | 0.007 | 0.54 | 0.018 | 0.31 | 0.039 | 0.13 | 0.020 | 0.29 | 0.019 | 0.30 |
|  | PMI | 0.003 | 0.70 | 0.008 | 0.49 | 0.004 | 0.63 | 0.002 | 0.74 | 0.006 | 0.55 | 0.001 | 0.82 |
|  | pH | 0.002 | 0.71 | 0.017 | 0.32 | 0.006 | 0.57 | 0.011 | 0.42 | 0.006 | 0.55 | 0.002 | 0.74 |
|  | DI | 0.001 | 0.84 | <0.001 | 0.95 | 0.015 | 0.51 | 0.016 | 0.51 | 0.024 | 0.42 | 0.013 | 0.55 |
|  | RIN | ***0.098*** | ***0.01*** | 0.061 | 0.06 | ***0.073*** | ***0.04*** | ***0.167*** | ***<0.001*** | ***0.072*** | ***0.04*** | 0.037 | 0.14 |
|  |  |  |  |  |  |  |  |  |  |  |  |  |  |
| BA 44 | Age | 0.016 | 0.34 | 0.046 | 0.10 | 0.016 | 0.34 | 0.023 | 0.25 | 0.050 | 0.09 | 0.020 | 0.29 |
|  | PMI | 0.018 | 0.31 | 0.010 | 0.44 | 0.018 | 0.31 | 0.011 | 0.43 | <0.001 | 0.90 | 0.019 | 0.29 |
|  | pH | **0.078** | **0.03** | 0.039 | 0.13 | ***0.091*** | ***0.02*** | ***0.079*** | ***0.03*** | 0.045 | 0.10 | 0.034 | 0.16 |
|  | DI | 0.004 | 0.75 | 0.013 | 0.55 | <0.001 | 0.99 | 0.006 | 0.69 | 0.019 | 0.47 | 0.005 | 0.72 |
|  | RIN | ***0.131*** | ***0.005*** | ***0.145*** | ***0.003*** | ***0.163*** | ***0.002*** | ***0.163*** | ***0.002*** | ***0.101*** | ***0.015*** | ***0.101*** | ***0.015*** |
