# Supplementary material for: Validating reference genes using minimally transformed qpcr data: findings in human cortex and outcomes in schizophrenia
Source: BMC Psychiatry. 2016 May 20;16:154. doi: 10.1186/s12888-016-0855-0 (PMC4875643; doi:10.1186/s12888-016-0855-0)
Supplement: Additional file 5: Figure S2. — The relationship between levels of mRNA and RNA Integrity Number (RIN) for PPIA (A) and TFB1M (B) as well as CNS pH and SKP1 (C) in BA 8 as well as GAPDH (D), SNCA (E), TFB1M (F) and NOL 9 (G) with RIN in BA 9. The linear regression line is shown ± 95 % prediction confidences. (DOCX 102 kb) [file 12888_2016_855_MOESM5_ESM.docx]

Supplementary Figure 2: The relationship between levels of mRNA and RNA Integrity Number (RIN) for *PPIA* (A) and *TFB1M* (B) as well as CNS pH and *SKP1* (C) in BA 8 as well as *GAPDH* (D), *SNCA* (E), *TFB1M* (F) and *NOL 9* (G) with RIN in BA 9. The linear regression line is shown ± 95% prediction confidences.
