# Supplementary material for: Validating reference genes using minimally transformed qpcr data: findings in human cortex and outcomes in schizophrenia
Source: BMC Psychiatry. 2016 May 20;16:154. doi: 10.1186/s12888-016-0855-0 (PMC4875643; doi:10.1186/s12888-016-0855-0)
Supplement: Additional file 6: Figure S3. — The relationship between levels of mRNA and CNS pH for GAPDH (A), SNCA (B) and NOL9 (C) as well as RNA Integrity Number (RIN) and GAPDH (D), PPIA (E), SNCA (F), NOL 9 (G), TFB1M (H) and SKP1 (I) in BA 44. The linear regression line is shown ± 95 % prediction confidences. (DOCX 131 kb) [file 12888_2016_855_MOESM6_ESM.docx]

Supplementary Figure 3: The relationship between levels of mRNA and CNS pH for *GAPDH* (A), *SNCA* (B) and *NOL9* (C) as well as RNA Integrity Number (RIN) and *GAPDH* (D), *PPIA* (E), *SNCA* (F), *NOL 9* (G), *TFB1M* (H) and *SKP1* (I) in BA 44. The linear regression line is shown ± 95% prediction confidences.
