# Supplementary material for: Validating reference genes using minimally transformed qpcr data: findings in human cortex and outcomes in schizophrenia
Source: BMC Psychiatry. 2016 May 20;16:154. doi: 10.1186/s12888-016-0855-0 (PMC4875643; doi:10.1186/s12888-016-0855-0)
Supplement: Additional file 7: Table S4. — A comparison of the mean standard deviation (Mean STD) for each potential reference gene expressed as a ratio of mRNA for one gene to all remaining genes in the three regions of the human cortex. STD = standard deviation of data for each individual gene. (DOCX 20 kb) [file 12888_2016_855_MOESM7_ESM.docx]

|  |  | BA8 |  |  |  | BA9 |  |  |  | BA44 |  |  |  |
| --- | --- | --- | --- | --- | --- | --- | --- | --- | --- | --- | --- | --- | --- |
|  |  | Mean ΔCt | STD | Mean STD | Rank | Mean ΔCt | STD | Mean STD | Rank | Mean ΔCt | STD | Mean STD | Rank |
| GAPDH vs | PPIA | 0.693 | 0.590 |  |  | 0.797 | 0.454 |  |  | 0.842 | 0.430 |  |  |
|  | SNCA | 4.229 | 0.553 |  |  | 4.290 | 0.464 |  |  | 4.403 | 0.454 |  |  |
|  | NOL9 | 8.387 | 0.739 |  |  | 8.268 | 0.527 |  |  | 8.332 | 0.621 |  |  |
|  | TFB1M | 7.768 | 0.436 |  |  | 7.525 | 0.466 |  |  | 7.851 | 0.509 |  |  |
|  | SKP1 | 12.590 | 1.281 | 0.720 | 4 | 11.520 | 0.873 | 0.557 | 5 | 12.030 | 0.731 | 0.549 | 2 |
|  |  |  |  |  |  |  |  |  |  |  |  |  |  |
| PPIA vs | GAPDH | 0.693 | 0.590 |  |  | 0.797 | 0.454 |  |  | 0.842 | 0.430 |  |  |
|  | SNCA | 3.536 | 0.517 |  |  | 3.493 | 0.404 |  |  | 3.561 | 0.448 |  |  |
|  | NOL9 | 7.695 | 0.789 |  |  | 7.472 | 0.545 |  |  | 7.490 | 0.502 |  |  |
|  | TFB1M | 7.076 | 0.473 |  |  | 6.728 | 0.323 |  |  | 7.009 | 0.417 |  |  |
|  | SKP1 | 11.900 | 1.103 | 0.694 | 3 | 10.72 | 0.872 | 0.520 | 2 | 11.190 | 0.832 | 0.525 | 1 |
|  |  |  |  |  |  |  |  |  |  |  |  |  |  |
| SNCA vs | GAPDH | 4.229 | 0.553 |  |  | 4.290 | 0.464 |  |  | 4.403 | 0.454 |  |  |
|  | PPIA | 3.536 | 0.517 |  |  | 3.493 | 0.404 |  |  | 3.561 | 0.448 |  |  |
|  | NOL9 | 4.159 | 0.674 |  |  | 3.978 | 0.443 |  |  | 3.929 | 0.463 |  |  |
|  | TFB1M | 3.540 | 0.498 |  |  | 3.234 | 0.438 |  |  | 3.448 | 0.633 |  |  |
|  | SKP1 | 8.361 | 1.068 | 0.662 | 1 | 7.231 | 0.827 | 0.515 | 1 | 7.631 | 0.767 | 0.553 | 3 |
|  |  |  |  |  |  |  |  |  |  |  |  |  |  |
| NOL9 vs | GAPDH | 8.387 | 0.739 |  |  | 8.268 | 0.527 |  |  | 8.332 | 0.621 |  |  |
|  | PPIA | 7.695 | 0.789 |  |  | 7.472 | 0.545 |  |  | 7.490 | 0.502 |  |  |
|  | SNCA | 4.159 | 0.674 |  |  | 3.978 | 0.443 |  |  | 3.929 | 0.463 |  |  |
|  | TFB1M | 0.619 | 0.747 |  |  | 0.744 | 0.511 |  |  | 0.481 | 0.673 |  |  |
|  | SKP1 | 4.202 | 1.368 | 0.864 | 5 | 3.253 | 0.746 | 0.555 | 4 | 3.703 | 0.912 | 0.634 | 5 |
|  |  |  |  |  |  |  |  |  |  |  |  |  |  |
| TFB1M vs | GAPDH | 7.768 | 0.436 |  |  | 7.525 | 0.466 |  |  | 7.851 | 0.509 |  |  |
|  | PPIA | 7.076 | 0.473 |  |  | 6.728 | 0.323 |  |  | 7.009 | 0.417 |  |  |
|  | SNCA | 3.540 | 0.498 |  |  | 3.234 | 0.438 |  |  | 3.448 | 0.633 |  |  |
|  | NOL9 | 0.619 | 0.747 |  |  | 0.744 | 0.511 |  |  | 0.481 | 0.673 |  |  |
|  | SKP1 | 4.821 | 1.253 | 0.682 | 2 | 3.997 | 0.898 | 0.527 | 3 | 4.184 | 0.879 | 0.622 | 4 |
|  |  |  |  |  |  |  |  |  |  |  |  |  |  |
| SKP1 vs | GAPDH | 12.590 | 1.281 |  |  | 11.520 | 0.873 |  |  | 12.030 | 0.731 |  |  |
|  | PPIA | 11.900 | 1.103 |  |  | 10.720 | 0.872 |  |  | 11.190 | 0.832 |  |  |
|  | SNCA | 8.361 | 1.068 |  |  | 7.231 | 0.827 |  |  | 7.631 | 0.767 |  |  |
|  | NOL9 | 4.202 | 1.368 |  |  | 3.253 | 0.746 |  |  | 3.703 | 0.912 |  |  |
|  | TFB1M | 4.821 | 1.253 | 1.215 | 6 | 3.997 | 0.898 | 0.843 | 6 | 4.184 | 0.879 | 0.824 | 6 |
|  |  |  |  |  |  |  |  |  |  |  |  |  |  |
|  |  |  |  |  |  |  |  |  |  |  |  |  |  |

Supplementary Table 4: A comparison of the mean standard deviation (Mean STD) for each potential reference gene expressed as a ratio of mRNA for one gene to all remaining genes in the three regions of the human cortex. STD = standard deviation of data for each individual gene.
