# Supplementary material for: Validating reference genes using minimally transformed qpcr data: findings in human cortex and outcomes in schizophrenia
Source: BMC Psychiatry. 2016 May 20;16:154. doi: 10.1186/s12888-016-0855-0 (PMC4875643; doi:10.1186/s12888-016-0855-0)
Supplement: Additional file 8: Table S5. — Genes suggested as most suitable to use as reference genes by three different algorithms. (DOCX 18 kb) [file 12888_2016_855_MOESM8_ESM.docx]

Supplementary Table 5: Genes suggested as most suitable to use as reference genes by three different algorithms.

|  | Software Ranking | 1 | 2 | 3 | 4 | 5 | 6 |
| --- | --- | --- | --- | --- | --- | --- | --- |
| Cortical Region | Software |  |  |  |  |  |  |
| Brodmann’s area 8 | BestKeeper  *Stability Factor* | *GAPDH*  *0.709* | *PPIA*  *0.727* | *SNCA*  *0.821* | *NOL9*  *0.920* | *TFB1M*  *1.006* | *SKP1*  *1.011* |
|  | NormFinder *Stability Factor* | *SNCA*  *0.209* | *PPIA*  *0.331* | *TFB1M*  *0.370* | *GAPDH*  *0.445* | *NOL9*  *0.673* | *SKP1*  *1.140* |
|  | geNorm  *M* | *GAPDH /*  *TFB1M*  *0.436* |  | *SNCA*  *0.496* | *PPIA*  *0.511* | *NOL9*  *0.602* | *SKP1*  *0.806* |
|  |  |  |  |  |  |  |  |
| Brodmann's area 9 | BestKeeper  *Stability Factor* | *GAPDH*  *0.539* | *PPIA*  *0.644* | *TFB1M*  *0.652* | *SNCA*  *0.801* | *NOL9*  *0.836* | *SKP1*  *1.050* |
|  | NormFinder *Stability Factor* | *SNCA*  *0.279* | *PPIA*  *0.313* | *TFB1M*  *0.332* | *NOL9*  *0.335* | *GAPDH*  *0.367* | *SKP1*  *0.779* |
|  | geNorm  *M* | *PPIA / TFB1M*  *0.323* |  | *SNCA*  *0.388* | *GAPDH*  *0.425* | *NOL9*  *0.458* | *SKP1*  *0.586* |
|  |  |  |  |  |  |  |  |
| Brodmann's area 44 | BestKeeper  *Stability Factor* | *GAPDH*  *0.684* | *TFB1M*  *0.705* | *PPIA*  *0.817* | *SNCA*  *0.923* | *SKP1*  *0.949* | *NOL9*  *1.022* |
|  | NormFinder *Stability Factor* | *PPIA*  *0.261* | *GAPDH*  *0.291* | *SNCA*  *0.309* | *TFB1M*  *0.456* | *NOL9*  *0.479* | *SKP1*  *0.740* |
|  | geNorm  *M* | *GAPDH / PPIA*  *0.429* |  | *SNCA*  *0.444* | *TFB1M*  *0.482* | *NOL9*  *0.515* | *SKP1*  *0.618* |
